# 

**Published:** 1889-09-28

**Authors:** 


					? 111 \z&. ^
tfo. 157,,-VolrfL
-September 28th, 188S.
WSSjjT It - -*8 Wr^K xrci- annum, on. on., yuou ixco.    , , .?
1\ IFI /M Monthly Parts, 6d.: post free 7id.
Registered at the (rentral Post OTlc# as a Newspaper.)
' * .J,

				

